# Differential proteomics study of postharvest *Volvariella volvacea* during storage at 4 °C

**DOI:** 10.1038/s41598-020-69988-8

**Published:** 2020-08-04

**Authors:** Lei Zha, Mingjie Chen, Changxia Yu, Qian Guo, Xu Zhao, Zhengpeng Li, Yan Zhao, Chuanhua Li, Huanling Yang

**Affiliations:** Institute of Edible Fungi, Shanghai Academy of Agricultural Sciences, Shanghai, 201403 China

**Keywords:** Fungal biology, Fungal genomics

## Abstract

The postharvest storage of *Volvariella volvacea* is an important factor limiting the industry development. Low-temperature storage is the traditional storage method used for most edible fungi, but *V. volvacea* undergoes autolysis at low temperature. To understand the molecular mechanism underlying the low-temperature autolysis of *V. volvacea* after harvesting, fruiting bodies of *V. volvacea* strain V23 were stored at 4 °C. Based on our previous study, in which the changes of morphological and physiological indexes during storage for 0, 6, 12, 24, 30, 36, 48 and 60 h were measured; four time points, namely, 0, 12, 24 and 60 h, were selected for this differential proteomics study. The proteomic changes in the postharvest storage samples were studied by isobaric tags for relative and absolute quantification-coupled two-dimensional liquid chromatography-tandem mass spectrometry (2D LC–MS/MS). A total of 2,063 proteins were identified, and 192 differentially expressed proteins (DEPs), including 24 up-regulated proteins and 168 down-regulated proteins, were detected after 12 h of storage. After 24 h of storage, 234 DEPs, including 48 up-regulated and 186 down-regulated proteins, were observed, and after 60 h, 415 DEPs, including 65 up-regulated proteins and 350 down-regulated proteins, were observed. An in-depth data analysis showed that the DEPs participated in various cellular processes, particularly metabolic processes. In this study, we combined Gene Ontology and Kyoto Encyclopedia of Genes and Genomes pathway analyses, and the results focused on oxidative phosphorylation and ubiquitin mediated proteolysis pathways. In addition, *sdh2*, *uba1* and *ubc1* was confirmed by quantitative real-time polymerase chain reaction, and the results showed that the expression of these genes were consistent with their protein level. Based on the literature and our results, it is speculated that the identified DEPs, *such as* ATP1, SDH2, COR1, UBA1, COX4, UBC1 and SKP1 play a key role in the low-temperature autolysis of *V. volvacea*.

## Introduction

*Volvariella volvacea* is a commercially important edible fungus cultivated in tropical and subtropical regions, particularly in Southeast Asian countries^1,2^. In recent years, the market demand for *V. volvacea* has rapidly increased due to its unique flavor^3^ and high nutritional value^4^. *V. volvacea* also has pharmaceutical value because it contains antitumor polysaccharides and immunomodulatory lectins^5–7^. *V. volvacea* is delicious and mainly sold fresh. In addition, this fungus exhibits rapid growth and development, has high respiratory intensity, and is easy-to-open umbrella at 28–35 °C, and these features are responsible for the loss of its edible value over a short period of time^8^. In addition, *V. volvacea* is native to tropical and subtropical regions and is thus sensitive to low temperature. The fungus is usually autolysed at 0–5 °C^9,10^, and this autolysis accelerates its decay and causes softening and liquefaction. Therefore, the postharvest storage problem of *V. volvacea* has become a bottleneck that restricts its commercial development^11^. In our previous study, changes in the color, weight loss rate, relative conductivity and malondialdehyde (MDA) content of *V. volvacea*, which are key indicators of its quality, were measured during the storage at 4 °C. The results showed that the weight loss rate (Fig. S1), relative conductivity (Fig. S2) and MDA content (Fig. S3) increased during storage, and the appearance (Fig. S4) of *V. volvacea* fruiting bodies began to liquefy after low-temperature damage^12^.

Although the low-temperature autolysis of *V. volvacea* has been studied extensively, the specific molecular mechanism is still unclear^13^. The observed changes in quality characteristics, such as texture and color, might be caused by the complex biochemical and physiological changes in these compounds during storage^14^. Based on these reasons, selecting an appropriate method is important for studying the autolysis of *V. volvacea*. The application of proteomics in life science research is helpful for understanding complex biological mechanisms and identifying biomarkers related to quality traits^15^. Previous proteomic studies have investigated various edible fungi, including *Lentinula edodes*^16^, *Boletus edulis*^17^, *Flammulina velutipes*^18^, *Pleurotus tuber-regium*^19^, *Termitomyces heimii*^20^, *Sparassis crispa*, and *Hericium erinaceus*^21^. To understand the internal mechanism of the low-temperature autolysis of the fruiting bodies of *V. volvacea*, we studied the changes in the proteomes of *V. volvacea* during exposure to low-temperature stress using the isobaric tags for relative and absolute quantification (iTRAQ) technique. We also investigated the transcription of *sdh2*, *uba1* and *ubc1* by quantitative real-time polymerase chain reaction (qRT-PCR). This study will provide new insights into the low-temperature autolysis of *V. volvacea* from a proteomic perspective.

## Materials and methods

### Sample collection

Fresh fruiting bodies of *V. volvacea* V23 (provided by Shanghai Fanshun Edible Fungus Professional Cooperative, Shanghai, China) were delivered to the laboratory within 25 min after harvest. We selected disease-free samples of uniform size with complete fruiting bodies and smooth surfaces, and these samples were randomly divided into four groups, with three replicates in each group. Each group was stored at 4 °C (low temperature) for 0, 12, 24 and 60 h (labeled as L0, L12, L24 and L60, respectively); these time points were selected because significant changes in phenotypic and physiological parameters^12^ were detected at these times by Zhao et al. All the samples were frozen with liquid nitrogen and kept at −  80 °C for protein extraction.

### Protein preparation^22^

The samples were ground with liquid nitrogen. One milliliter of phenol extract was then added, and the mixture was mixed well. The same volume of a phenol-Tris-HCl (pH 7.5) saturated solution was then added, and the resulting mixture was incubated at 4 °C for 30 min with shaking and inverted several times during the incubation. The phenolic upper layer was collected after centrifugation for 10 min at 4 °C and 7,100×*g*. A fivefold volume of precooled 0.1 M ammonium acetate–methanol solution was added, and the mixture was incubated overnight at − 20 °C to induce precipitation. The samples were then centrifuged for 10 min at 4 °C and 12,000×*g*, and the precipitate was collected. A fivefold volume of precooled methanol was added, and the mixture was lightly mixed to clean the sample. The sample was then centrifuged for 10 min at 4 °C and 12,000×*g*, and the precipitate was collected. The same above-described process was then repeated with the following exceptions: the methanol was replaced with acetone, and operation were repeated to remove the remaining methanol. The sample was then centrifuged for 10 min at 4 °C and 12,000×*g*, and the precipitate was collected and dried at room temperature (5 min). The sample was dissolved in a pyrolysis solution and incubated at room temperature for 3 h. The solution was centrifuged at room temperature for 10 min at 12,000×*g*, and the supernatant was collected and centrifuged again. The supernatant included the total protein from the tissue. The protein concentration was determined using the Bradford method and then stored at − 80 °C for further analysis.

### Protein digestion and iTRAQ labeling^23^

One hundred micrograms of sample was added to 10 K ultrafiltration tubes, and 120 μL of reductant buffer (10 mM DTT, 8 M urea, and 100 mM triethyl ammonium bicarbonate (TEAB), pH 8.0) was added to the sample. The mixture was then incubated for 1 h, and iodoacetamide (IAA) was added to a final concentration of 50 mM. The sample was incubated at room temperature for 40 min and centrifuged at 12,000 rpm and 4 °C for 20 min, and the bottom solution in the collection tube was discarded. Subsequently, 100 μL of sample was added to 100 μL of 300 mM TEAB buffer. The solution was centrifuged at 12,000 rpm for 20 min; these steps were repeated twice. A new collection tube was prepared by adding 100 μL of 300 mM TEAB buffer and 2 mL of 1 μg/μL sequencing trypsin solution to an ultrafiltration tube and incubating for 12 h at 37 °C. The peptide segment was centrifuged at 12,000 rpm for 20 min, and the enzymatically hydrolyzed peptide segment was collected. Subsequently, 50 μL of 200 mM TEAB buffer was added to the ultrafiltration tube, and the mixture was then centrifuged at 12,000 rpm for 20 min. The solution at the bottom of the tube was collected and lyophilized. The freeze-dried samples were labeled in a 1.5-mL Ep tube by adding 100 μL of 200 mM TEAB buffer to the mix with an eddy current. The iTRAQ reagent (ABI, Foster City, CA) was equilibrated at room temperature and centrifuged to the bottom of the tube. Two hundred microliters of isopropanol was then added, and the mixture was mixed with an eddy current and centrifuged; these steps were repeated once. The L0, L12, L24 and L60 samples were labeled with 113, 114, 115 and 116 tags, respectively. One hundred microliters of iTRAQ reagent was added to the sample, and an eddy current was applied. The sample was mixed well and placed at room temperature for 2 h. Subsequently, 200 μL of water was added to stop the reaction, and the mixture was allowed to sit for 30 min. The sample was then freeze-dried and stored at − 80 °C.

### RPLC analysis

For reversed-phase liquid chromatography (RPLC) analysis^23^, RP separation was performed using an 1100 HPLC system (Agilent Technologies, Santa Clara, CA, USA) with an Agilent Zorbax Extend RP column (5 µm, 150 mm × 2.1 mm). Mobile phases A (2% acetonitrile in HPLC-grade water) and B (98% acetonitrile in HPLC-grade water) were used for the RP gradient. The solvent gradient was as follows: 0–8 min, 98% A; 8.00–8.01 min, 98–95% A; 8.01–38 min, 95–75% A; 38–50 min, 75–60% A; 50–50.01 min, 60–10% A; 50.01–60 min, 10% A; 60–60.01 min, 10–98% A; and 60.01–65 min, 98% A. The tryptic peptides were separated at a fluid flow rate of 300 µL/min and monitored at 210 and 280 nm. The dried samples were harvested from 8 to 50 min, and elution buffer was collected from the pipeline at 1-min intervals into sample tubes numbered from 1 to 10. The separated peptides were lyophilized for MS detection.

### MS analysis

All the analyses were performed using a Triple TOF 5600 mass spectrometer (AB SCIEX, Framingham, MA, USA) equipped with a NanoSpray III source (AB SCIEX, Framingham, MA, USA). The samples were loaded onto a capillary C18 trap column (3 cm × 100 µm) and then separated by a C18 column (15 cm × 75 µm) on an Eksigent nanoLC-1D plus system (AB SCIEX, Framingham, MA, USA). The flow rate was 300 nL/min, and the linear gradient was set as follows: 0–0.5 min, 95–92% A; 0.5–48 min, 92–74% A; 48–61 min, 74–62% A; 61–61.1 min, 62–15% A; 61.1–67 min, 15% A; 67–67.1, 15–95% A; and 67.1–70 min, 95% A. Mobile phases A and B consisted of 2% Acetonitrile (ACN)/0.1% Formic acid (FA) and 95% ACN/0.1% FA, respectively.

The data were acquired with an ion spray voltage of 2.4 kV, a curtain gas pressure of 35 psi, a nebulizer gas pressure of 5 psi, and an interface heater temperature of 150 °C. The MS scan was performed between 400 and 1,500 with an accumulation time of 250 ms. For IDA, 30 MS/MS spectra (80 ms each, mass range of 100–1,500) of MS peaks with an intensity higher than 260 and a charge state between 2 and 5 were acquired. A rolling collision energy voltage was used for CID fragmentation to acquire the MS/MS spectra. The mass was dynamically excluded for 22 s.

### Protein identification and quantification

All the Triple TOF 5600 MS/MS raw data were thoroughly searched against the sample protein database using ProteinPilot software (v.5.0) (AB SCIEX, Framingham, MA, USA). And the *V. volvacea* V23 database (https://mycocosm.jgi.doe.gov/Volvo1/Volvo1.home.html) was used for analysis. ITRAQ 8-plex was selected for protein quantification. A global false discovery rate (FDR) of < 1% was used, and the peptide groups with at least two peptides were considered for quantification.

### Bioinformatics and statistical analysis

For characterization of the DEP properties, Gene Ontology (GO) enrichment and Kyoto Encyclopedia of Genes and Genomes (KEGG) pathway enrichment analyses were performed using the OmicsBean Group Data Integration Analysis Cloud Platform (https://www.omicsbean.cn/).

### RNA extraction, reverse transcription, and quantitative real-time polymerase chain reaction analysis

The total RNA of each sample was extracted with the RNAiso Plus Kit (Takara, Shiga, Japan). The genomic DNA in the sample was removed using the Prime Script RT Reagent Kit with gDNA Eraser (Takara, Shiga, Japan) and the total RNA was reversely transcribed. qRT-PCR was performed using a TB green Premier Ex *Taq* Kit (Takara, Shiga, Japan). We selected MSF1-domain-containing protein (*msf1*) as the reference gene of *V. volvacea* to quantitatively analyze the expression of target genes^24^. The NCBI prime design tool (https://www.ncbi.nlm.nih.gov/tools/prime-blast) was used to design the primers, and the primer data are summarized in Table 1. The relative expression levels of the target genes were calculated using the 2^−ΔΔCt^ method^25^.

**Table 1:** Primers used in this study.

ID	Primer	Sequence (5′ to 3′)
1	Vvo-*sdh2*-F	GATGAGCCAGCGAACAAG
2	Vvo-*sdh2*-R	AGAGGGTCAGGTCGGGTA
3	Vvo-*uba1*-F	TGATTGGTGGCTTTGTTG
4	Vvo-*uba1*-R	GGTTGGCATTCTTCTTCTG
5	Vvo-*ubc1*-F	AAGTCGCCAAGCATTACA
6	Vvo-*ubc1*-R	TTCCTCAACTACACGGTCA
7	Vvo-*msf1*-F	TCTGTCGACCCCACAACTGGCATAA
8	Vvo-*msf1*-R	TCTGTGTAGCTGGGTCGACGAATGA

## Results

### Identification of reliable proteins and DEPs

**Figure 1:**
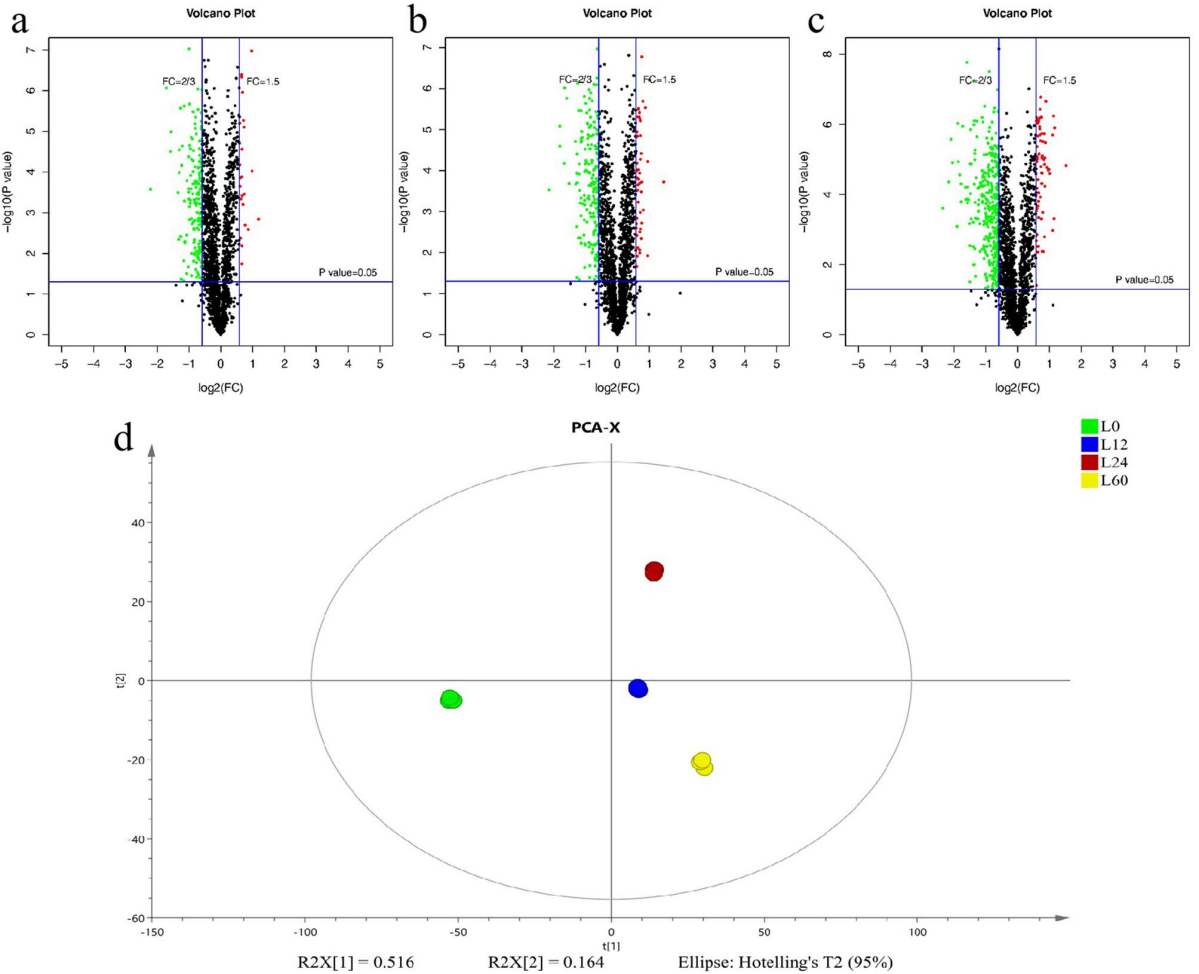
(**a**–**d**) Volcano plots showing DEPs in the *V. volvacea* groups L12-L0 (**a**), L24-L0 (**b**) and L60-L0 (**c**). The red (FC ≥ 1.5) and green (FC ≤ 2/3) dots indicate proteins that were significantly altered in terms of abundance *p* ≤ 0.05). (**d**) Principle component analysis (L0, L12, L24 and L60).

**Figure 2:**
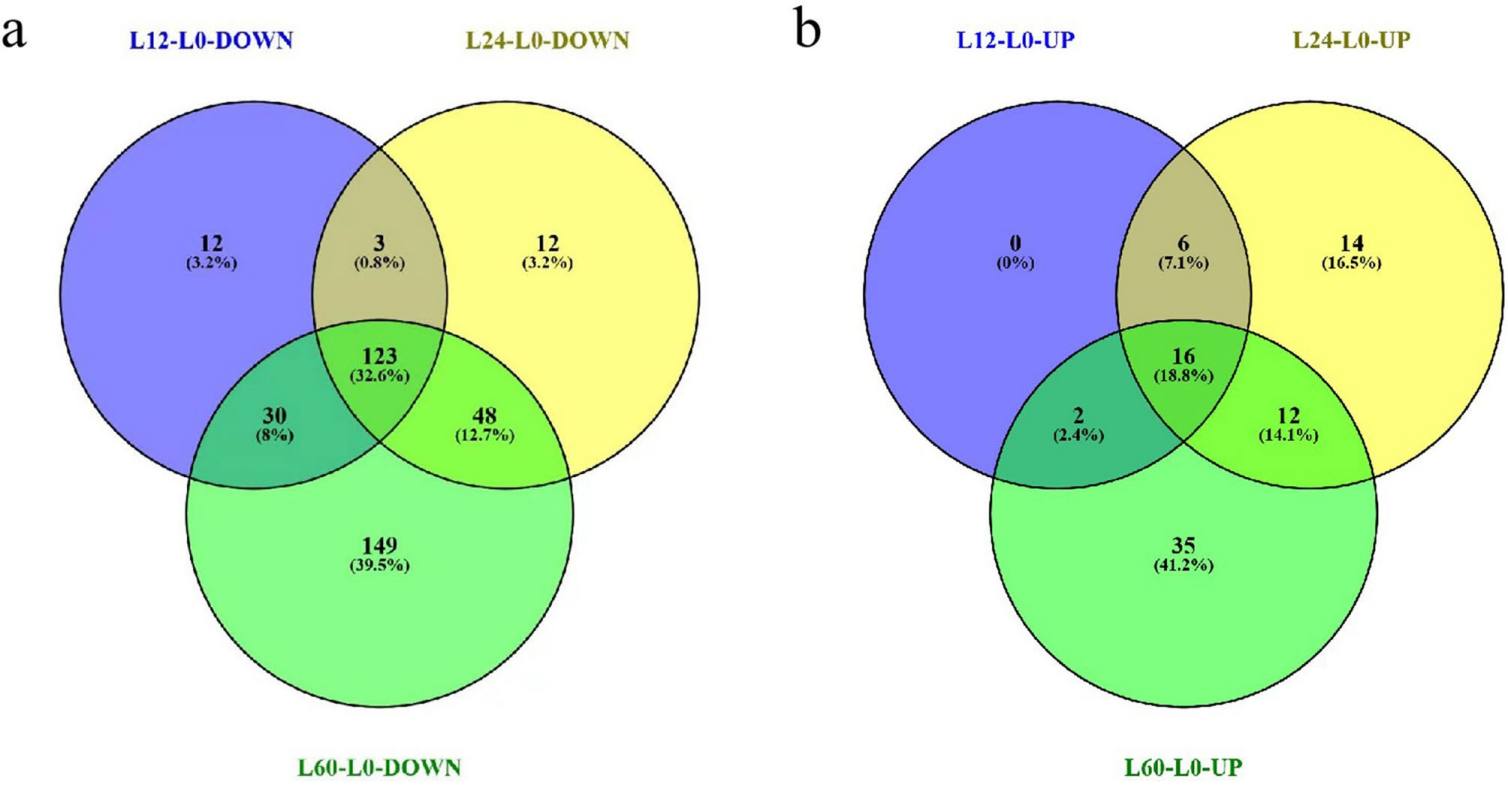
(**a**, **b**) Venn diagrams showing the overlap in differentially expressed proteins among the three *V. volvacea* comparisons (L12-L0, L24-L0 and L60-L0). The numbers of DEPs that were down-regulated (**a**) and up-regulated (**b**) during *V. volvacea* storage at 4 ºC are shown.

The quantitative proteomic analysis of *V. volvacea* storage at 4 °C identified a total of 2063 authentic proteins. The following criteria were used to identify the significant DEPs: fold change (FC) > 1.5 or FC < 2/3 and *p* value < 0.05. A total of 462 DEPs were obtained, and the number of DEPs increased significantly during storage (Fig. 1a–c); specifically, the number of DEPs with respect to the control group reached 192, 234 and 415 after storage for 12, 24 and 60 h, respectively. A total of 139 common DEPs were detected among the groups (Fig. 2a, b). In addition, 24, 48 and 65 upregulated DEPs and 168, 186 and 350 downregulated DEPs were identified after storage for 12, 24 and 60 h, respectively (Table 2). Additionally, the levels of the DEPs (including upregulated and downregulated proteins) increased as the storage time increased, which indicated that the proteome changed significantly during storage. In addition, the results showed significant separation between the L0, L12, L24 and L60 datasets (Fig. 1d). The 12 samples in this analysis were divided into four main groups, with three replicates in each group.

**Table 2:** Effects of storage at 4 °C on the protein profile of *V. volvacea.*

Number	Comparison group	Total DEPs	Up-regulated DEPs	Down-regulated DEPs
1	L12/L0	192	24	168
2	L24/L0	234	48	186
3	L60/L0	415	65	350

### Hierarchical cluster analysis

**Figure 3:**
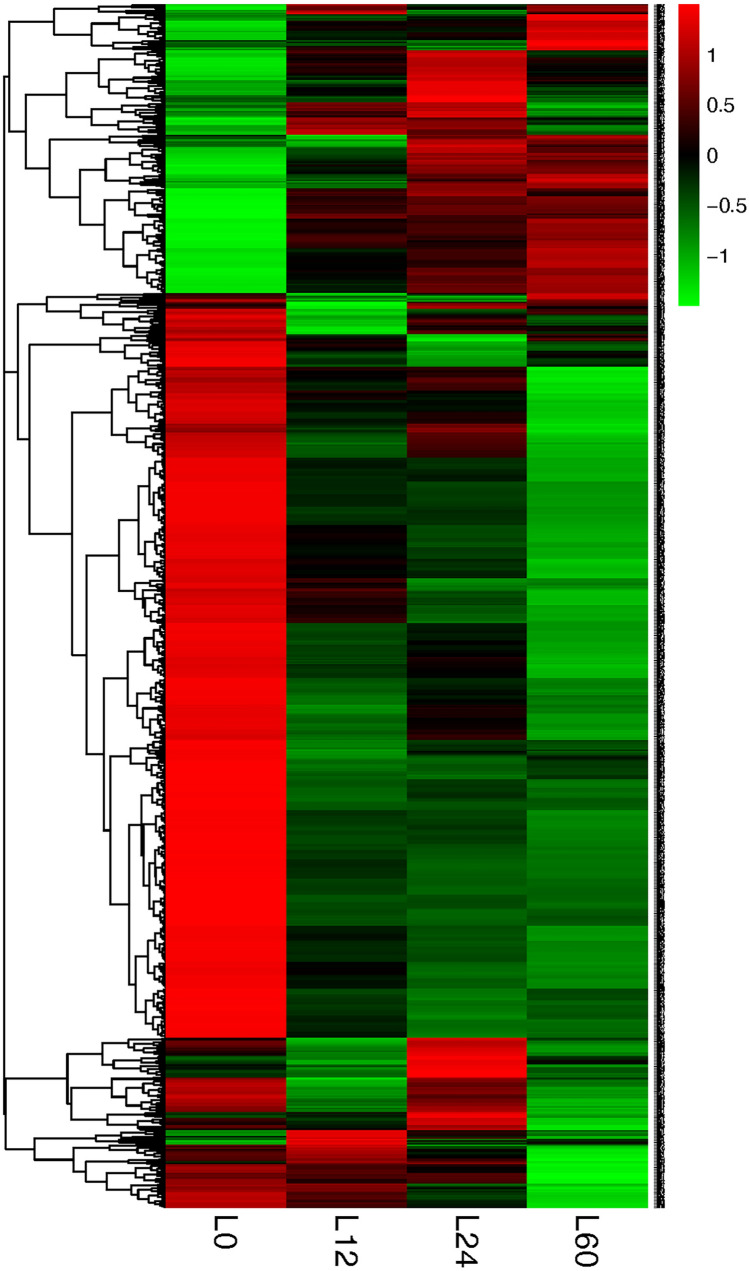
Cluster analysis of *V. volvacea* DEPs in the L12, L24 and L60 groups compared with the L0 group. The red, green, and black color indicated increases, decreases, and no changes in protein abundance, respectively.

Hierarchical cluster analysis uses algorithms to infer a system of relationships, and thus group individual proteins or groups^26^. A hierarchical cluster analysis of the DEPs was performed to better visualize the differences in protein abundance among the four groups, namely, L0, L12, L24, L60 (Fig. 3). Compared to control group L0, the analysis of the up-regulated and down-regulated proteins in the L12, L24 and L60 groups showed that both the upregulated and downregulated proteins significant changes as the storage time increased. The hierarchical cluster analysis results were consistent with the morphological and physiological changes observed in the *V. volvacea* fruiting bodies.

### GO enrichment analysis of the DEPs

**Figure 4:**
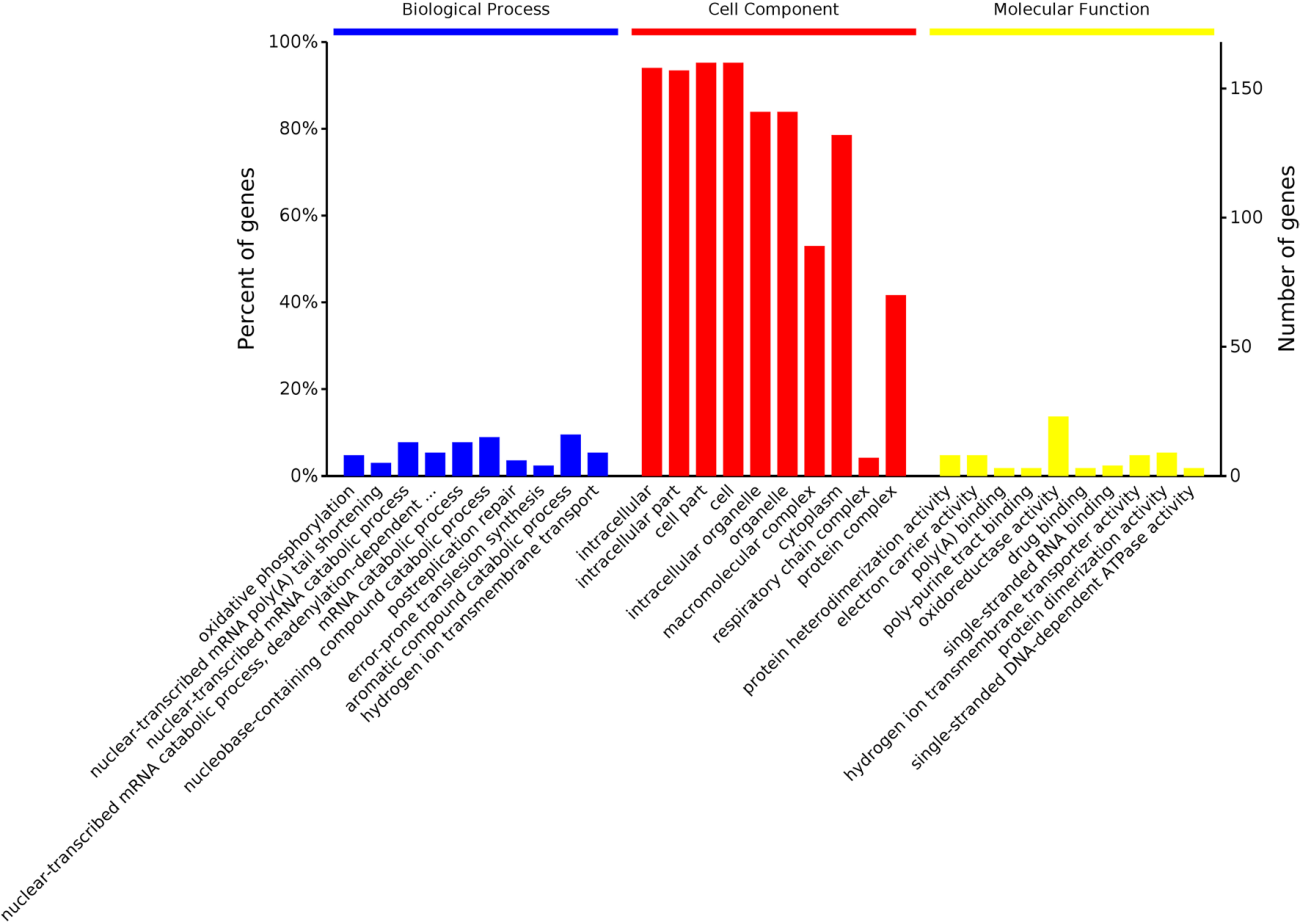
GO enrichment analysis of the DEPs identified in the L12-L0 comparison.

**Figure 5:**
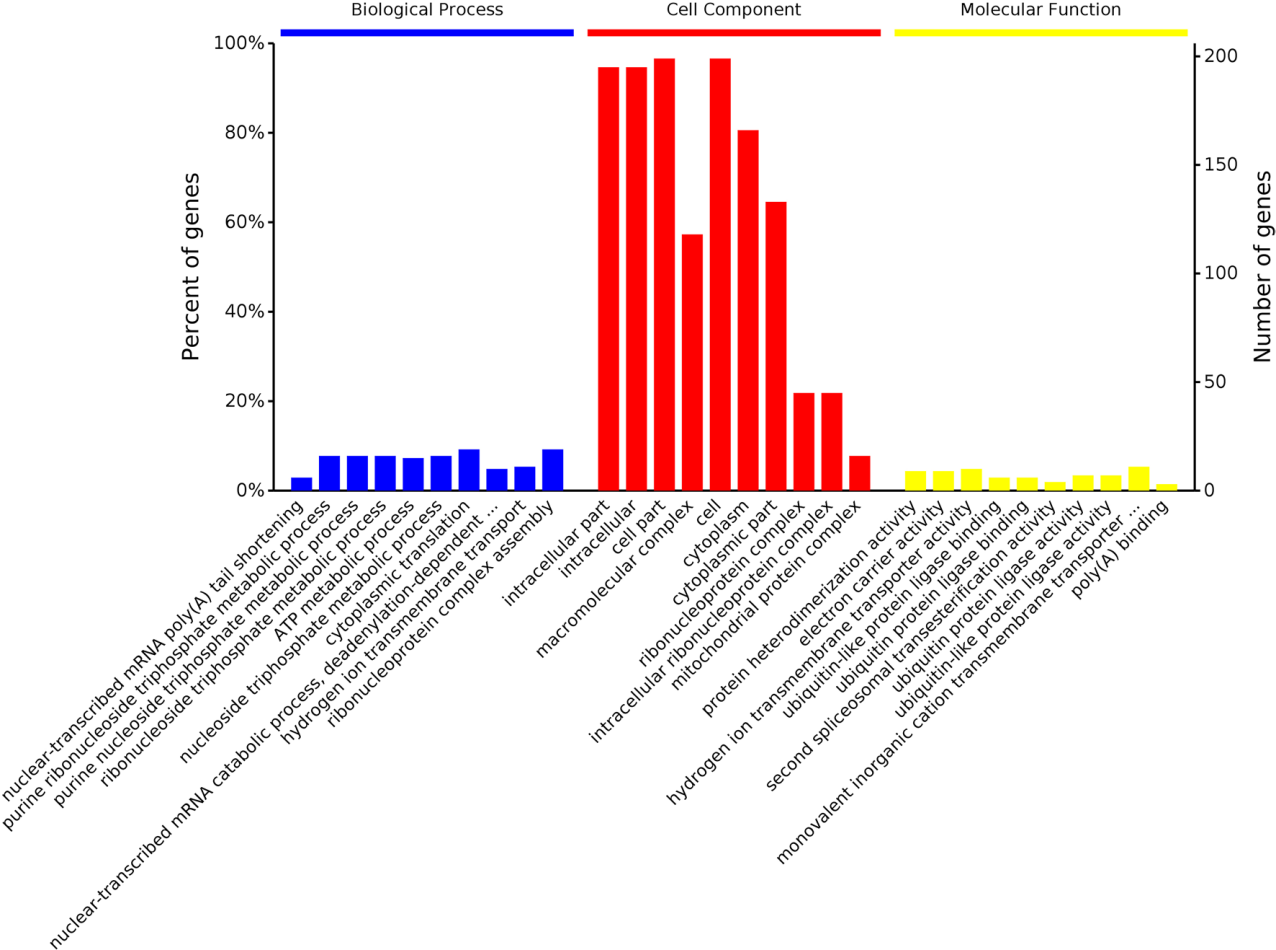
GO enrichment analysis of the DEPs identified in the L24-L0 comparison.

**Figure 6:**
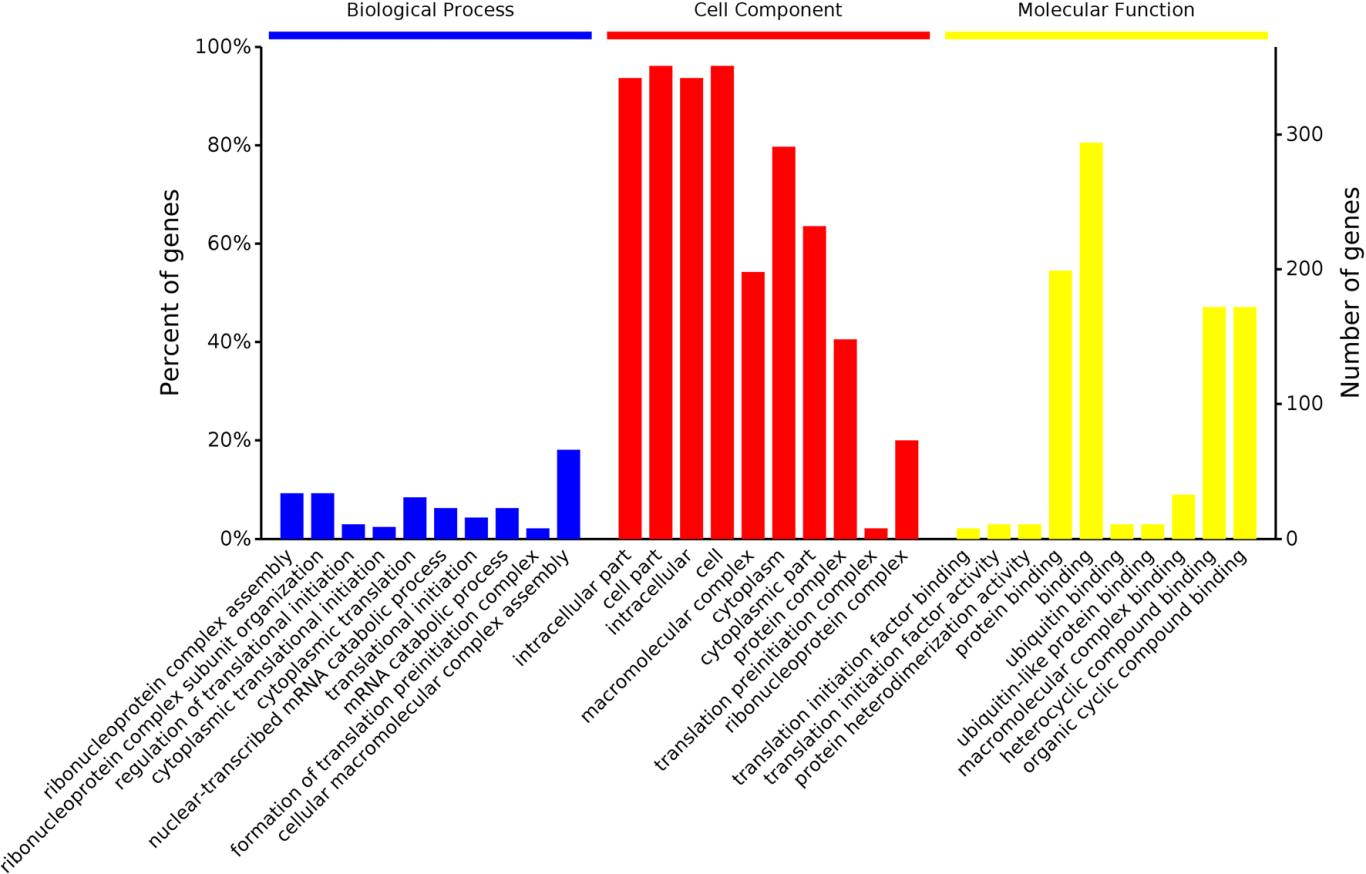
GO enrichment analysis of the DEPs identified in the L60-L0 comparison.

GO is an internationally standardized tool for evaluating functional classifications that comprehensively describing the properties of genes and proteins in organisms. GO uses three broad categories that describe the constituent cell components (CCs), the molecular functions (MFs) of a gene or protein, and the biological processes (BPs) in which a gene participate^27^. The GO results showed that the DEPs identified from the comparison of the L12 group with the L0 group (Fig. 4) were mainly involved in cellular component organization or biogenesis, metabolic processes, biological processes, and single-organism processes. The DEPs identified in the comparison of the L24 group with the L0 group (Fig. 5) were mainly involved in cellular component organization or biogenesis, metabolic processes, biological regulation, cellular processes, single-organism processes, and other biological processes, and the DEPs obtained from the comparison of the L60 group with the L0 group (Fig. 6) were mainly involved in cellular component organization or biogenesis, metabolic processes, biological regulation, cellular processes, response to stimulus, localization, and single biological processes, such as organism processes.

The GO results from the comparison of the L12-L0 groups showed that the location of the DEPs mainly included cell parts, intracellular parts, macromolecular complexes, organelle parts and other cellular components. Additionally, the location of the DEPs identified from the L24-L0 comparison mainly included cell parts, macromolecular complexes, cells, organelles, organelle parts and other cellular components. The location of the DEPs obtained from the L60-L0 comparison mainly included cell parts, cells, macromolecular complexes, organelles, organelle parts, cellular and membrane parts and other cellular components.

### Biological pathway enrichment analysis of the DEPs

**Figure 7:**
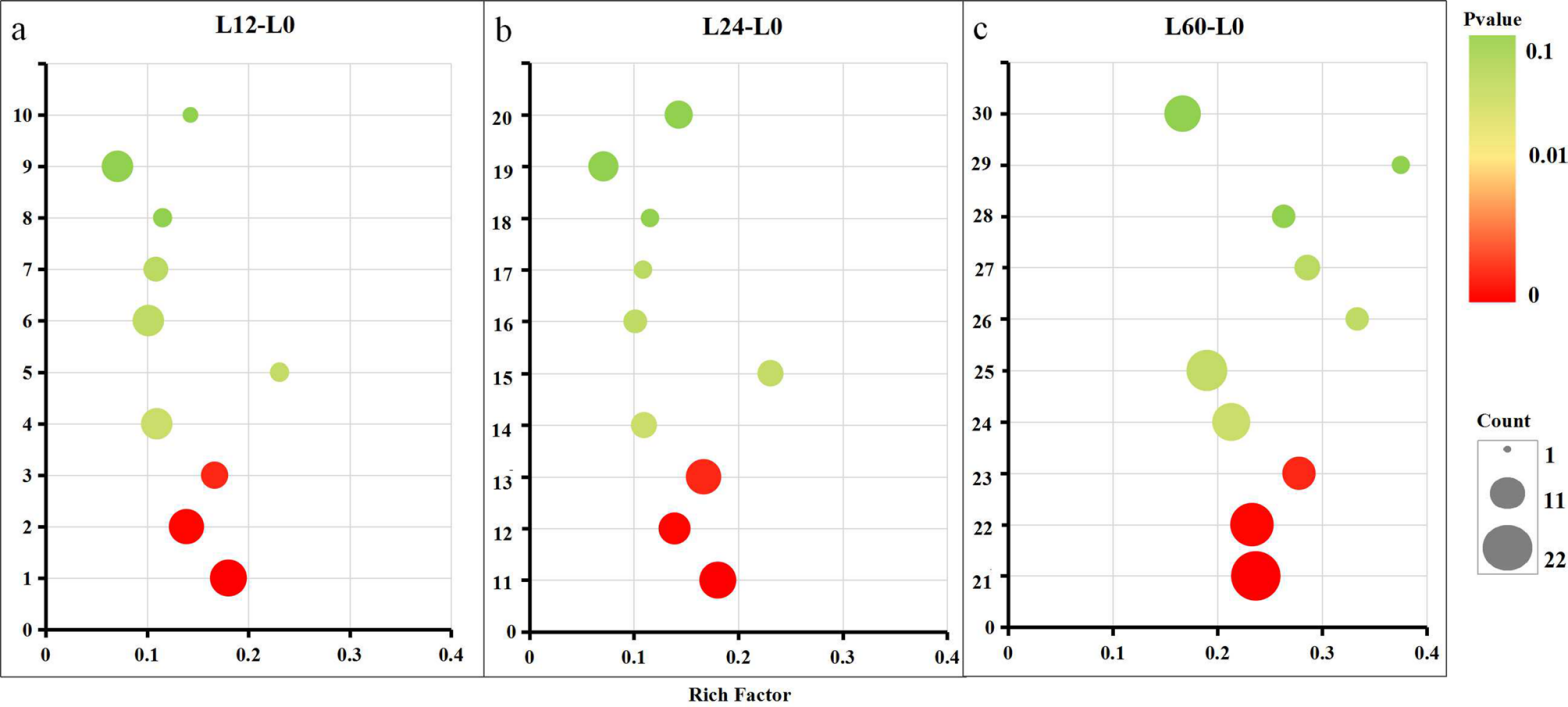
(**a**–**c**) Bubble diagrams of the top 10 KEGG pathways enriched in the DEPs obtained from the L12-L0 (**a**), L24-L0 (**b**), and L60-L0 (**c**) comparisons. The numbers in the Y-axis represent the different KEGG pathways: (1) RNA degradation, (2) oxidative phosphorylation, (3) phagosome, (4) pyrimidine metabolism, (5) β-alanine metabolism, (6) spliceosome, (7) mRNA surveillance pathway, (8) glycerolipid metabolism, (9) MAPK signaling pathway-yeast, (10) tyrosine metabolism, (11) oxidative phosphorylation, (12) RNA degradation, (13) RNA transport, (14) mRNA surveillance pathway, (15) ubiquitin mediated proteolysis, (16) phagosome, (17) insulin resistance, (18) tryptophan metabolism, (19) spliceosome, (20) endocytosis, (21) RNA transport, (22) endocytosis, (23) phagosome, (24) RNA degradation, (25) spliceosome, (26) lysine degradation, (27) SNARE interactions in vesicular transport, (28) fatty acid degradation, (29) sulfur relay system, and (30) oxidative phosphorylation. The rich factor represents the ratio of the DEPs to all annotated proteins enriched in the pathway. The bubble scale represents the number of DEPs, and the depth of the bubble color represents the adjusted *p* value.

The KEGG database is an online database of metabolic pathways, enzymatic pathways, enzymes, and biochemicals that integrates genomic, chemical and system functional information genes^28^. The KEGG pathway database contains molecular interaction networks of specific biological pathways and specific changes in specific organisms and can be used to determine the most important signal transduction pathways and biochemical metabolic pathways in which a protein participates. In vivo, different proteins coordinate and regulate each other in biological processes, and pathway-based analysis is thus helpful for understanding the biological functions of genes or proteins. In this study, we mainly analyzed the top 10 enriched pathways (Fig. 7).

The KEGG enrichment analysis of the DEPs identified from the L12-L0, L24-L0, and L60-L0 comparisons showed that the RNA degradation and phagosome pathways reached significance. Additionally, the number of proteins enriched in the RNA degradation and phagosome pathways also increased during prolonged storage. In addition, the oxidative phosphorylation pathway was significantly enriched in the DEPs identified from the L12-L0 and L24-L0 comparisons, and the amount of enriched DEPs increased during storage. The ubiquitin-mediated proteolysis pathway was significantly enriched in the DEPs identified in the L24-L0 comparison, and the amounts of enriched DEPs increased with increases in the storage time. Pathways such as fatty acid degradation and lysine degradation were not significantly enriched in the DEPs obtained from the L12-L0 or L24-L0 comparisons but were significantly enriched in the DEPs identified in the L60-L0 comparison. These results indicated that the RNA degradation, phagosome and oxidative phosphorylation, fatty acid degradation, lysine degradation and ubiquitin-mediated proteolysis pathways in *V. volvacea* were all significantly affected by the low-temperature conditions. The functions of these DEPs and specific pathways are discussed further in the next section.

### Quantitative real-time polymerase chain reaction analysis

**Figure 8:**
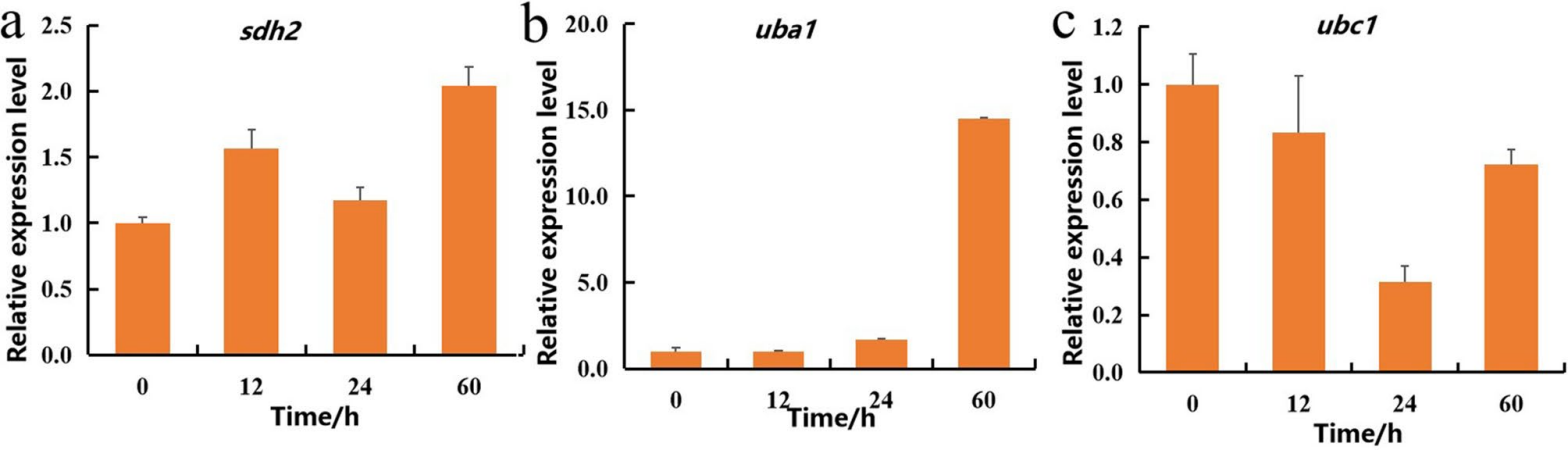
(**a**–**c**) qRT-PCR validation results of key proteins: *sdh2* (**a**), *uba1* (**b**), and *ubc1* (**c**).

Western blotting is the best approach for the verification of proteomics data^29^, but few specific antibodies are currently available for proteins of *V. volvacea*. In this study, the expression of *sdh2*, *uba1* and *ubc1* was confirmed by quantitative real-time polymerase chain reaction (qRT-PCR). The results showed that the relative expression of *uba1* increased with increases in the storage time (Fig. 8b). The relative expression of *sdh2* after storage was higher than that in L0, but the relative expression of this gene in L24 was lower than that in L12 (Fig. 8a). The relative expression of *ubc1* in the stored samples was lower than that in L0, but the relative expression of this gene in L60 was higher than that in L24 (Fig. 8c). These results are not exactly consistent with the proteomics findings, which might be the result of protein degradation and gene modification. However, *sdh2* and *uba1* showed an upward trend, whereas *ubc1* showed a downward trend, which was consistent with the trend obtained at the protein level.

## Discussion

**Figure 9:**
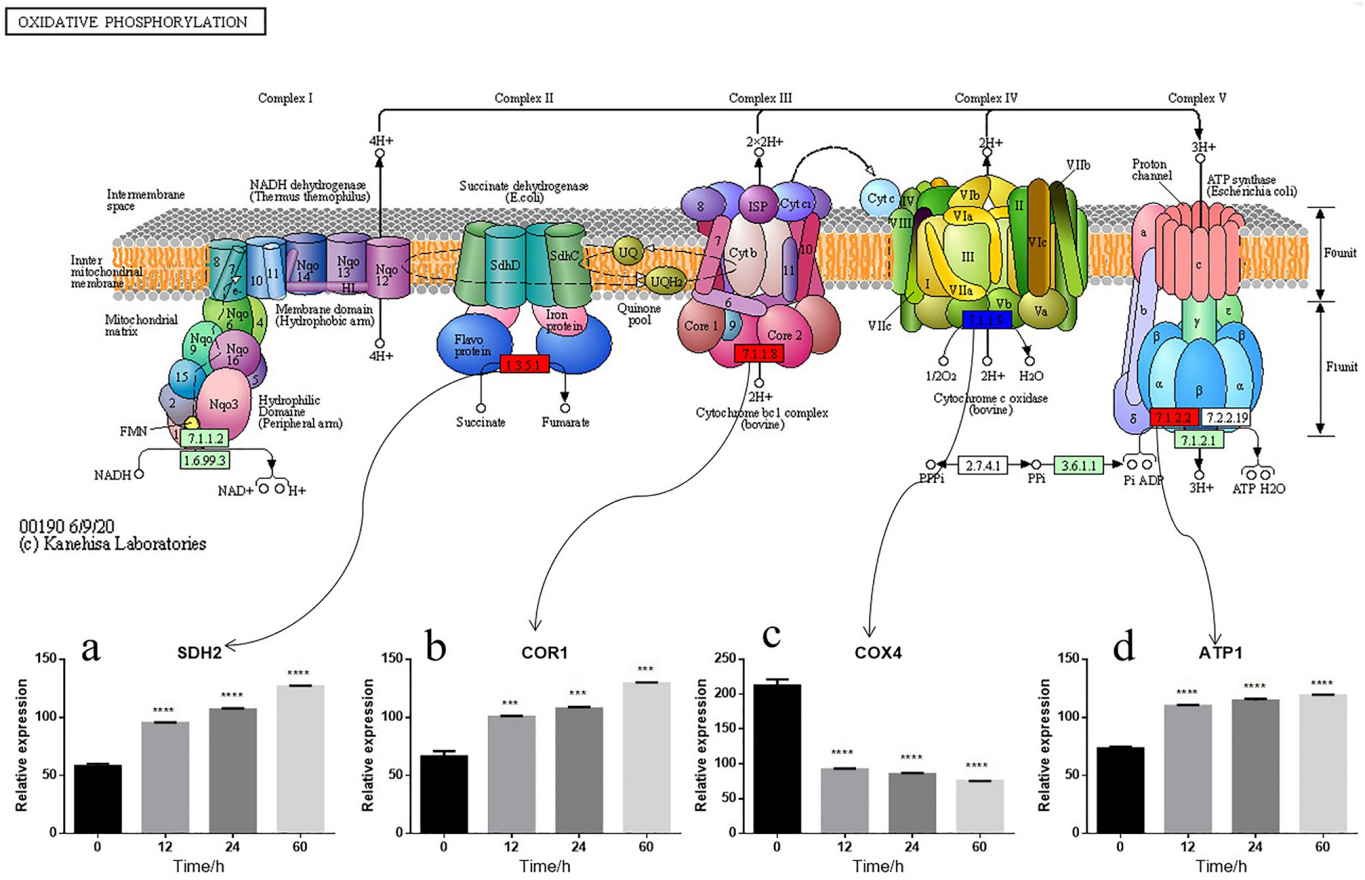
(**a**–**d**) Representative metabolic pathway maps of DEPs involved in oxidative phosphorylation pathway in KEGG^30^ (including the expression of SDH2 (**a**), COR1 (**b**), COX4 (**c**) and ATP1 (**d**) proteins during the storage of *V. volvacea* at 4 °C).

**Figure 10:**
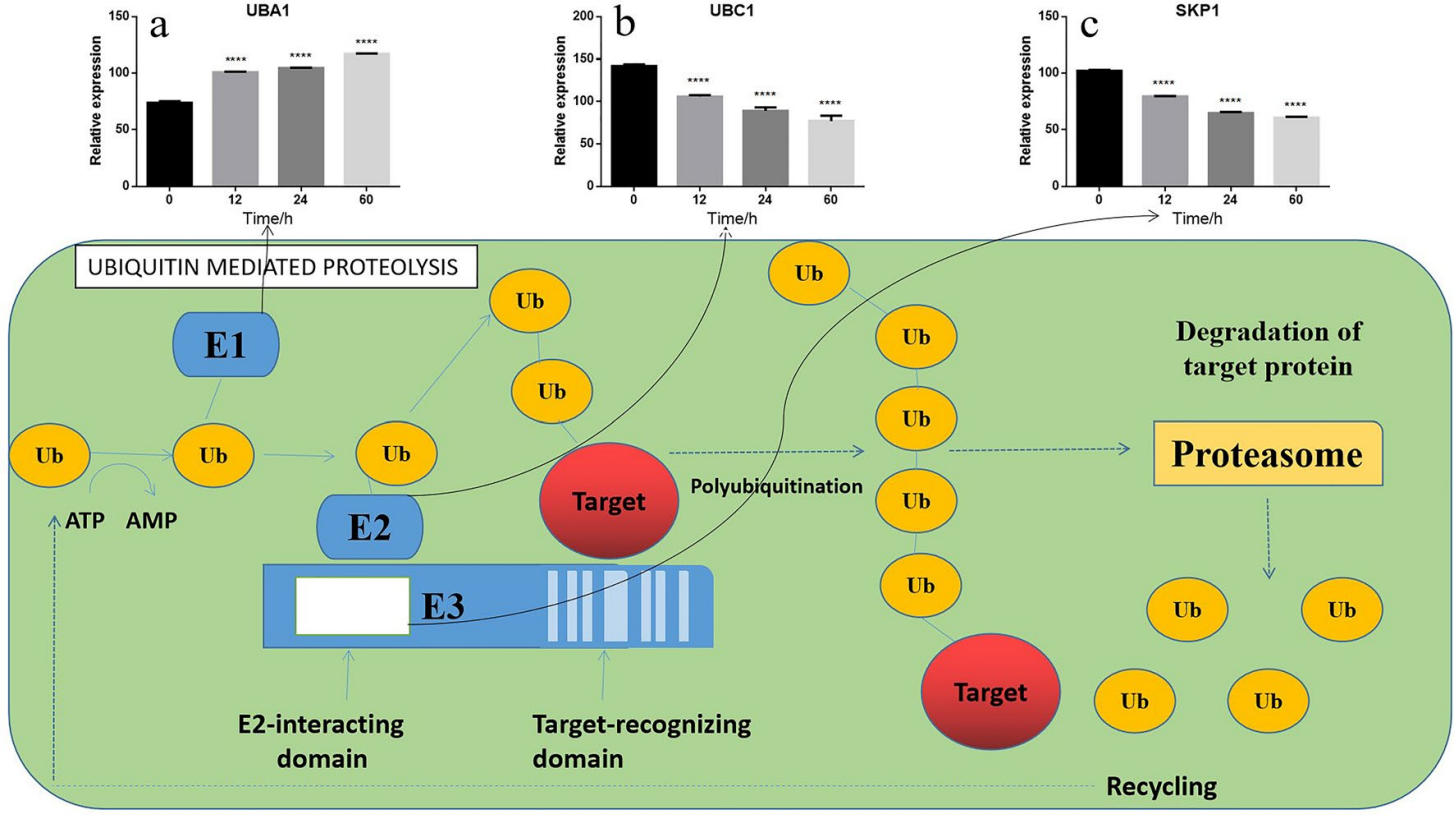
(**a**–**c**) Representative metabolic pathway maps of DEPs involved in ubiquitin mediated proteolysis pathway in KEGG (including the expression of UBA1 (**a**), UBC1 (**b**) and SKP1 (**c**) proteins during the storage of *V. volvacea* at 4 °C).

In this study, the experimental results identified DEPs involved in multiple pathways. Based on the results from the statistical data analysis combined with our biological research objectives, we selected the oxidative phosphorylation (Fig. 9) and ubiquitin mediated proteolysis pathways (Fig. 10) for discussion.

In this study, we identified DEPs that involved in oxidative phosphorylation (ID: sce00190), which is a pathway related to energy metabolism. The proteomic results showed that the abundances of ATP1, SDH2 and COR1 proteins increased significantly during storage at 4 °C. Additionally, the COX4 protein abundance significantly decreased during storage at 4 °C. ATP1 is the α subunit of mitochondrial ATPase F1^31^. Some studies have reported that a decrease in ATP1 will lead to decreases in ATPase synthesis, ATPase activity, ATP production and the energy supply^32^. In this study showed that ATP1 was significantly up-regulated, which might be due to the response to low-temperature stress in terms of the energy supply. SDH is a marker for the localization of the mitochondrial electron transfer enzyme respiratory chain and mitochondrial membrane^33^ and plays an important role in aerobic respiratory metabolism^34^. In addition, SDH can be used as an index for evaluating the levels of the tricarboxylic acid cycle^35^, changes in which can reflect the functional state of mitochondria^36^, and is an important respiratory enzyme^33^. The iron sulfur substituents encoded by SDH2 contain ubiquinone-binding sites, which transfer the hydrogen removed from succinic acid to ubiquinone for the generation of ubiquinone^32^. Few studies have investigated SDH2 under stress, but its expression affects energy production. The results showed that the expression of ATP1 (Fig. 9d) and SDH2 (Fig. 9a) in *V. volvacea* increased with prolonged storage time, and this increased expression might elevate the level of oxidative phosphorylation and provide sufficient energy to *V. volvacea* cells to improve their adaptation to a low-temperature environment.

The ubiquinol-cytochrome c reductase complex (complex III or cytochrome b-c1 complex) forms part of the mitochondrial respiratory chain and generates an electrochemical potential coupled to ATP synthesis; briefly, this complex couples electron transfer from ubiquinol to cytochrome c. COR1 might mediate the formation of a complex between cytochromes c and c1^37^. Previous studies have found that many plants, including *Arabidopsis thaliana*, are highly resistant to freezing when exposed to low and nonfreezing temperatures. This response, termed cold acclimation, is associated with the induction of *cor* (cold-regulated) genes mediated by the DNA regulatory C-repeat/drought-responsive element (CRT/DRE). In *Arabidopsis*, increased expression of CBF1, a transcriptional activator that binds to the CRT/DRE sequence, induces *cor* gene expression and increases the freezing tolerance of non-acclimated Arabidopsis plants^38,39^. It has speculated that the expression of the *cor1* gene in *V. volvacea* after low-temperature treatment might be related to the low-temperature tolerance. In addition, it has been reported that the *cor1* gene might be involved in the response to low-temperature stress^11,40^, such as by blocking changes in membrane lipids and increasing the levels of sugars and soluble proteins, which effectively prolongs the survival time of the mycelia of *V. volvacea* at 4 °C^40^. Newly synthesized proteins might enter or be adsorbed through the surface membrane, alter the stability of the membrane, or be distributed into the intercellular space, which would prevent or accelerate the cooling process^41^. The results of this study showed that COR1 (Fig. 9b) protein expression in *V. volvacea* was up-regulated during exposure to cold stress. This result further proves that *cor1* is involved in the response of *V. volvacea* to low-temperature stress at both the gene^11^ and protein levels and provides a theoretical reference for future improvements in *V. volvacea*.

Cytochrome c oxidase (COX, EC 1.93.1), which is the most important enzyme (complex IV) in the mitochondrial respiratory chain, catalyzes the transfer of electrons from cytochrome c to oxygen^42^. The nuclear-encoded cytochrome c oxidase subunit 4 (COX4), which is one of the key subunits, is very important for the stability of cytochrome c oxidase^43–45^. The COX4 protein abundance (Fig. 9c) decreased significantly during storage at 4 °C, which might inhibit the transfer of electrons from cytochrome c to oxygen during oxidative phosphorylation.

In this study, we found that the ubiquitin mediated proteolysis (ID: sce04120) pathway was also altered during cold storage, which is consistent with the results from previous studies on *V. volvacea*^46^. Ubiquitin mediated proteolysis is the most common function after protein ubiquitination and involves various aspects of cellular physiological processes, such as cell cycle regulation, cell differentiation, cell response to stress, and regulation of repair at the transcriptional level. Previous studies have found that higher plants can produce various proteins^47^ in response to high temperature, low temperature, drought and other forms of stresses. The ubiquitin–proteasome pathway (UPP) can efficiently and selectively degrade some proteins in cells, either partially or completely, and is involved in the intrinsic physiological regulation of various metabolic processes^48^. When *V. volvacea* was stored for 60 h at 4 °C, only the UBA1 (Fig. 10a) protein exhibited significantly increased levels among the DEPs involved in the pathway of interest. The main function of UBA1 is to catalyze the degradation of ubiquitin-binding-labeled cell proteins through the ubiquitin–proteasome system^49^. Ubiquitin activator (E1) has strong catalytic ability. In most species, only one enzyme is needed to activate the whole ubiquitination process^50^. Zhang screened the whole genome of *Saccharomyces cerevisiae* and found ubiquitin activator as a new autophagy regulatory protein^51^. Studies have also shown that ube2 is significantly up-regulated in *V. volvacea* (ubev2) under low-temperature stress^46^. In this study, the expression of UBA1 protein was elevated with the increases in the storage time. The qRT-PCR results also showed that the expression of the *uba1* gene after storage was higher than that in the control group. It has been speculated that the main role of UBA1 up-regulation during the storage of *V. volvacea* might be to accelerate the ubiquitin–proteasome system-catalyzed degradation of ubiquitin-binding-labeled cell proteins.

Moreover, the abundances of the SKP1 (Fig. 10c) and UBC1 (Fig. 10b) proteins significantly decreased during *V. volvacea* storage at 4 °C. These proteins are also involved in the Ubiquitin mediated proteolysis pathway and modify other proteins^52^. SKP1 is a core subunit of the SCF (SKP1/Cullin1/F-box protein) E3 ubiquitin ligase, which directs the ubiquitination of targets as a signal for 26S proteasome degradation. SKP1 is an adapter that links CUL1 and the F-box protein^53,54^. In a previous study, researchers identified potato virus X (PVX) triple gene block protein 3 (TGBp3)-induced SKP1 as a prosurvival factor or a defense-related factor^55,56^. In *Arabidopsis* and *Nicotiana benthamiana* plants, SKP1 contributes to auxin signaling, host defense against polerovirus infection^57–59^. These results suggest that SKP1 regulates cellular function and the levels of F-box protein by stabilizing its active form. Ubiquitin (Ub)-conjugating enzyme (UBC, E2) receives Ub from Ub-activating enzyme (E1) and transfers it to target proteins, and as a result, this enzyme plays a key role in Ub/26S proteasome-dependent proteolysis. UBC is reportedly involved in the tolerance of plants to abiotic stresses, including drought, salt, osmotic and water stress^60^. Previous studies have shown that the tolerance of *S. cerevisiae* to thermal stress might be regulated by dephosphorylation of the MAP kinase serine residues of UBC1 during exposure to this stress, which leads to increased tolerance to thermal stress^61^. Increases in *UBC1* expression and proteasome activity elevated cadmium (Cd) tolerance by reducing the Cd levels through the regulation of Cd transporter expression in *NtUBC1* tobacco^60^. Combined with those from previous studies, these results indicate that SKP1 and UBC1 are related to stress defense. The expression of SKP1 and UBC1 decreased with an increase in the storage time at low temperature in this study. At the same time, UBA1 is an ubiquitin-activating enzyme of ubiquitin mediated proteolytic pathway^62^, and its expression level increased during storage. Based on this, it is speculated that this may lead to Ubiquitin mediated proteolysis pathway accelerating protein degradation.

## Conclusion

In this study, iTRAQ technology was used to study the differentially expressed proteins in the fruiting bodies of *V. volvacea* stored at 4 °C for different time periods. The differential proteome results obtained from the L12-L0, L24-L0 and L60-L0 comparisons showed that oxidative phosphorylation and ubiquitin-mediated proteolysis pathways played important roles during the storage of *V. volvacea* stored at 4 °C. Some key proteins, such as the up-regulated proteins ATP1, SDH2, COR1 and UBA1 and the down-regulated proteins COX4, UBC1 and SKP1, may be associated with the adaptation of *V. volvacea* to low-temperature stress. These findings provide valuable evidence for a better understanding of the molecular mechanism underlying the low-temperature autolysis of *V. volvacea*.

## Supplementary information

Supplementary Information.
