# Supplementary material for: Differential proteomics study of postharvest Volvariella volvacea during storage at 4 °C
Source: Sci Rep. 2020 Aug 4;10:13134. doi: 10.1038/s41598-020-69988-8 (PMC7403728; doi:10.1038/s41598-020-69988-8)
Supplement: Supplementary file 1 — Supplementary Information. [file 41598_2020_69988_MOESM1_ESM.pdf]

Differential Proteomics Study of Postharvest *Volvariella volvacea* during Storage at 4 °C

Lei Zha†, Mingjie Chen†, Changxia Yu, Qian Guo, Xu Zhao, Zhengpeng Li, Yan Zhao*, Chuanhua Li, Huanling Yang

Institute of Edible Fungi, Shanghai Academy of Agricultural Sciences, Shanghai 201403, China

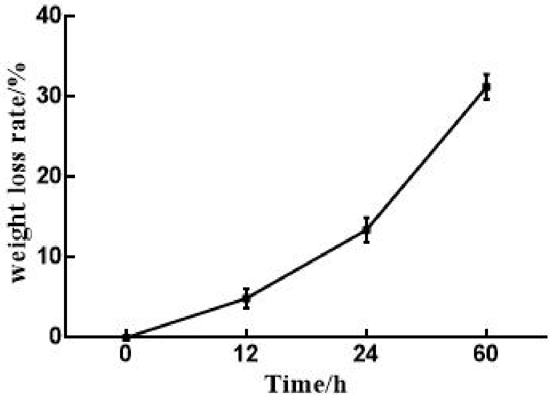

Figure S1. Weight loss rate of *Volvariella volvacea* fruiting bodies stored at 4 ℃

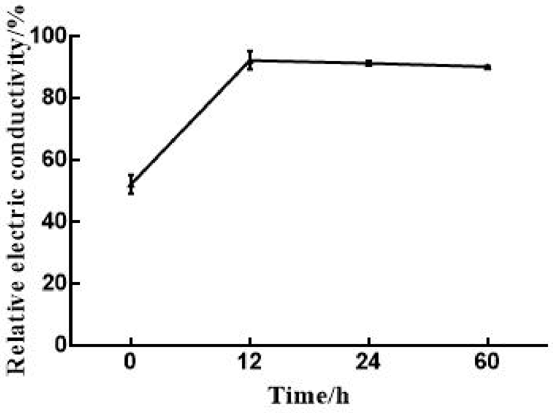

Figure S2. Relative electric conductivity of *Volvariella volvacea* fruiting bodies stored at 4 ℃

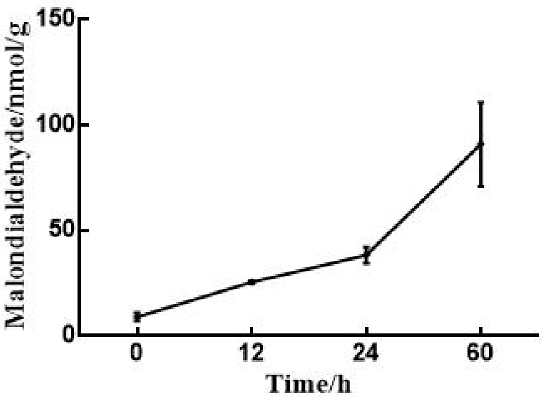

Figure S3. Malondialdehyde content of *Volvariella volvacea* fruiting bodies stored at 4 ℃

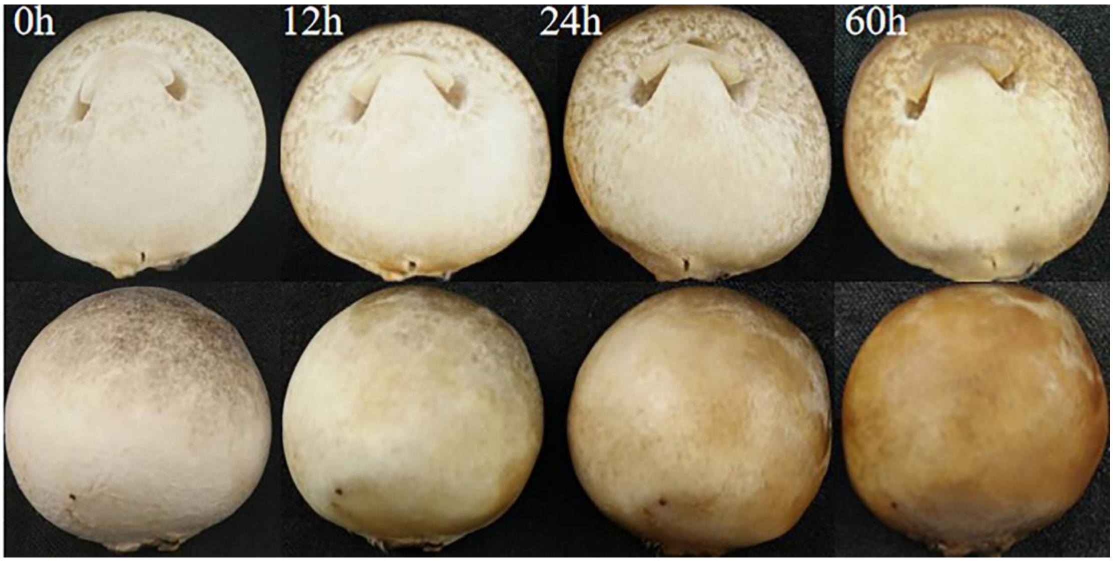

Figure S4. Sensory quality of *Volvariella volvacea* fruiting bodies stored at 4 ℃
